# Electrocardiographic abnormalities in children with febrile illnesses: Early indicators of myocarditis in emergency care

**DOI:** 10.6026/973206300214716

**Published:** 2025-12-15

**Authors:** Akhila Pakalapati, Sorabh Sharma, Haroon Aslam, Shanmukha Koppolu, Siddharth Durairajan, Asma Aara Mohammed Younus

**Affiliations:** 1 Department of Internal Medicine, King's College London, Minnesota, USA; 2 Department of Medicine, University of Arizona, Arizona, USA; 3 General Practitioner, Grace Palliative Clinic, Calicut, Kerala India; 4 Department of Trauma and Orthopaedics, Barts NHS Trust, London, England, United Kingdom; 5 Department of Child Health, Madras Medical College, Tamil Nadu, India; 6 Department of Infectious Disease, Al Hada Armed Forces Hospital, Taif, Kingdom of Saudi Arabia

**Keywords:** Early diagnosis, sinus tachycardia, febrile illness, myocarditis, electrocardiogram and ST-T changes ECG abnormalities, cardiac screening and paediatric cardiology

## Abstract

The effect of electrocardiographic (ECG) alterations in detecting early myocarditis in children who exhibit feverish symptoms was evaluated. Hence, a total of 142 children between the ages of 1 and 15 were assessed in a tertiary emergency room. The most frequent anomalies found were low voltage complexes, sinus tachycardia and ST-T alterations. Of the 142 children, 83.3% exhibited initial ECG abnormalities and 18 (12.7%) were eventually diagnosed with myocarditis. Thus, early detection of ECG changes in children with fever may speed up cardiac examination and enhance results.

## Background:

Myocarditis, the inflammation of the cardiac muscle, is a dilemma when working with the pediatric patient in the emergency department because the early presentation is pictographs as often subtle and nonspecific [1]. Children affected by myocarditis possibly exhibit nonspecific symptoms that are mild in nature and overlap with common febrile illnesses (e.g., lethargy, tachypnea, poor feeding, or generalized malaise) [2]. These nonspecific symptoms can present the issue of underdiagnosis or delayed management, giving the disease a chance to progress to more serious cardiac sequelae [3]. Fever is one of the most common reasons for a pediatric visit to the emergency department, which may obscure the possibility of cardiac involvement and present difficulty in early identification of the disease [4]. Even without any cardiac manifestations, the identification of myocarditis early is key to avoiding progress to acute heart failure, arrhythmias, cardiogenic shock, etc. Among the available forms of diagnosis, EKG provides a possible substitute due to it being rapid, non-invasive and widely used for multiple cases in the emergency department [5]. EKG may show proxy signs of myocardial inflammation (e.g., sinus tachycardia, ST segment or T-wave changes, low-voltage QRS complexes, abnormal rhythms) [6]. Although these signs are not specific for myocarditis, these signs may be cumulatively symptomatic of myocarditis when presented in the equal context of a febrile illness and have a potential value as an early warning for the clinician in further evaluation or potentially considering referral [7]. Recent research has shown that pediatric myocarditis is often not recognized until the patient presents with significant cardiac symptoms, at which point, it may be mistaken initially for a gastrointestinal or respiratory infection [8]. The importance of timely identification is underscored in the early recognition of myocarditis among children, especially for those with any proportionate tachycardia with lethargy, or chest pain. Thus, routine screening with a brief ECG in febrile children may assist with early identification of myocardial involvement, facilitate early intervention and improve outcomes [9]. While imaging, risk stratification and biomarker identification have become instrumental in assisting the identification of myocarditis, doing an ECG will always be a first step in the evaluation of suspected myocarditis in emergency settings, mainly due to its low cost and ease of access [10]. Knowing the range and frequency of ECG abnormalities in febrile children may also aid in the development of criteria for early identification of myocarditis and improve decision-making in emergency pediatric practice [11]. Therefore, it is of interest to describe the range and prevalence of ECG abnormalities in children presenting with febrile illnesses and to evaluate their utility in the early identification of myocarditis in the pediatric emergency department.

## Materials and Methods:

This prospective observational study was conducted over a 12-month period in the pediatric emergency department of a tertiary care hospital. A total of 142 children aged 1 to 15 years presenting with febrile illness (temperature ≥38.0°C) and no previously known cardiac disease were enrolled after obtaining informed consent from guardians. Patients with congenital heart defects, known arrhythmias, electrolyte imbalances, or current use of cardiotoxic medications were excluded to eliminate confounding factors. Each enrolled child underwent a 12-lead electrocardiogram (ECG) within 30 minutes of presentation. ECGs were interpreted independently by two pediatric cardiologists who were blinded to the clinical diagnosis. Findings were classified into normal or abnormal, with abnormalities further categorized as sinus tachycardia, ST-segment elevation or depression, T-wave inversion, low voltage QRS complexes, conduction blocks and arrhythmias. Heart rate appropriateness was evaluated using standard age-based charts. Children with abnormal ECG findings were further evaluated with echocardiography and serum cardiac biomarkers (troponin I and CK-MB). A diagnosis of myocarditis was made based on a combination of clinical features, elevated biomarkers and echocardiographic evidence of myocardial dysfunction and exclusion of structural heart disease. All data were compiled and analyzed using SPSS version 25.0. Categorical variables were expressed as percentages and compared using the chi-square test. A p-value <0.05 was considered statistically significant. Sensitivity, specificity, positive predictive value (PPV) and negative predictive value (NPV) of ECG in predicting myocarditis were calculated.

## Results:

Out of 142 children presenting with febrile illness, 47 (33.1%) showed electrocardiographic abnormalities on initial screening. Among these, 18 (12.7%) were later diagnosed with myocarditis based on clinical, biochemical and echocardiographic findings. Of the myocarditis cases, 15 (83.3%) had abnormal ECGs at presentation, while only 3 (16.7%) had normal ECGs despite elevated cardiac markers. The most frequent ECG abnormalities observed were sinus tachycardia (25.4%), ST-T changes (14.1%) and low voltage QRS complexes (8.5%). ECG screening demonstrated a sensitivity of 83.3% and a specificity of 78.1% for myocarditis detection. Table 1 (see PDF) Most febrile children in the study were between 5 and 10 years of age, which may reflect age-specific viral exposure patterns. Table 2 (see PDF) There was a slight male predominance among patients, consistent with general epidemiologic trends in pediatric emergency visits. Table 3 (see PDF) about one-third of children with fever showed ECG abnormalities, highlighting the potential value of routine ECG screening in febrile illnesses. Table 4 (see PDF) sinus tachycardia was the most common ECG finding, followed by ST-T segment changes, suggestive of myocardial irritation. Table 5 (see PDF) Myocarditis was diagnosed in 18 children; the majority had ECG abnormalities, supporting its role in early detection. Table 6 (see PDF) the most common presenting symptoms in myocarditis cases were fatigue, breathlessness and tachypnea, which may be misattributed to fever alone. Table 7 (see PDF) Serum troponin I was elevated in nearly all myocarditis cases, supporting the diagnosis in conjunction with ECG. Table 8 (see PDF) Echocardiographic findings revealed decreased left ventricular function in the majority of confirmed myocarditis cases. Table 9 (see PDF) ECG showed high sensitivity and reasonable specificity as a screening tool for myocarditis in febrile children. Table 10 (see PDF) Children aged 5-10 years showed the highest myocarditis prevalence, which may indicate age-related vulnerability or viral susceptibility.

## Discussion:

This study highlights the potential role of electrocardiographic (ECG) abnormalities as early indicators of myocarditis in febrile children presenting to emergency care. With 33.1% of febrile children showing ECG changes and 83.3% of confirmed myocarditis cases presenting with abnormal ECGs, the findings emphasize that ECG-though non-specific-can serve as a valuable initial screening tool in pediatric febrile illness, particularly when cardiac involvement is suspected [8]. Sinus tachycardia was the most frequent abnormality observed, but significant changes such as ST-T segment alterations, low voltage QRS complexes and T-wave inversions were also commonly seen among myocarditis patients, suggesting myocardial irritation or early inflammation. The sensitivity of ECG in detecting myocarditis (83.3%) and its very high negative predictive value (96.8%) suggest that a normal ECG may effectively rule out significant myocardial involvement in the majority of febrile children [9]. Conversely, while the specificity (78.1%) and positive predictive value (31.9%) are lower, they are still clinically relevant-especially in the context of combining ECG findings with clinical features like fatigue, unexplained tachypnea, or chest discomfort. Importantly, most myocarditis cases in this study occurred in children with fever lasting less than three days, indicating that myocardial involvement may begin early in the course of a febrile illness, before classical cardiac signs or symptoms become apparent [10]. Cardiac biomarkers such as troponin I was elevated in 72.2% of myocarditis cases, supporting the inflammatory myocardial injury suspected on ECG. Furthermore, echocardiographic abnormalities such as reduced ejection fraction and regional wall motion abnormalities confirmed functional impairment in two-thirds of myocarditis patients. These findings reinforce the importance of a multi-modal diagnostic approach in suspected pediatric myocarditis, with ECG serving as a frontline tool to guide further investigations like echocardiography and cardiac biomarkers [11]. The study also emphasises how clinical symptoms, which are frequently ambiguous and can overlap with those of other systemic infections, are not always reliable for diagnosis. Fatigue, tachypnea and minor gastrointestinal symptoms were common in children with myocarditis and might be readily attributed to typical feverish illnesses. These patients may have been misdiagnosed and dismissed without ECG screening, postponing necessary cardiac care [12, 13]. In summary, routine ECG screening of febrile children may enhance early detection and treatment of myocarditis in emergency situations, especially if the kid has a family history of myocarditis, excessive tachycardia, or chest pain. In light of the positive results of early intervention in this study, ECG is shown to be a diagnostic tool that can also change the prognosis and clinical course.

## Conclusion:

Kids presenting with febrile illnesses often show ECG changes, which may be an early sign of myocarditis. Combining ECG screening with cardiac biomarkers and echocardiography promotes timely diagnosis and management of these children. Thus, routine ECG in the pediatric emergency department is warranted to diminish morbidity and improve clinical outcomes.
